# Practices, perspectives, and psychological wellbeing among South Asian family caregivers in the US

**DOI:** 10.1002/alz70858_107725

**Published:** 2025-12-26

**Authors:** Sudev Namboodiri, Nirveen Kaur, Arveen Kaur, Jagnoor Randhawa, Puneet Kaler, Alyssa Weakley

**Affiliations:** ^1^ University of California, Davis School of Medicine, Sacramento, CA, USA

## Abstract

**Background:**

South Asian (SA) family caregivers living in the U.S. are understudied despite being a fast‐growing population with a disproportionate risk of Alzheimer's disease. The primary aim of this study was to characterize care practices, preferences, and psychological wellbeing among SA caregivers providing care to older generation family members. Given the collectivist nature of SA cultures, we expected most caregivers to live with their care partners and prefer similar, family‐based care for their future.

**Method:**

Community outreach and an Asian American caregiver registry (CARES) were used for recruitment. Participants completed questionnaires asking about caregiving practices (e.g., frequency, involvement), perspective on their personal future care (evaluated on a 1‐5 scale where 1=strongly agree and 5=strongly disagree), and caregiver stress/strain (Zarit Burden Interview).

**Result:**

Participants included 14 SA caregivers (age *M* = 49.5, *SD* = 15.5; education *M* = 17.1, *SD* = 2.3,). Seventy‐one percent identified as 1^st^ generation immigrant, 21% as 2^nd^ generation, and 7% did not specify. Most caregivers provided care to parents/in‐laws (79%). Common tasks requiring assistance were transportation (100% of caregivers surveyed) medical appointments (93%), cooking and medication management (71% for both). Seventy‐one percent provided physical support, whereas 57% provided support for cognitive limitations. Regarding future care, 43% of participants want to live with their family (non‐spouse) in the future, and this desire was significantly correlated with currently co‐residing with their care partner (*r* = ‐.68, *p* = .01). Sixty‐four percent indicate they would like to live alone/with spouse and 54% desire to not live in a retirement community. Mild to moderate levels of stress/strain were endorsed overall (*M* = 14.2, *SD* = 9.4), and were significantly correlated with a future care preference of living alone/with spouse (*r* = ‐.820, *p* < .001).

**Conclusion:**

In our sample, SA caregivers primarily live with the older generation family member they care for. Although less than half of the participants desire to live with family beyond their spouse in the future, currently providing in‐home care increased likelihood of wanting similar care. Importantly, caregiver stress/strain strongly increased desire to live alone/with spouse. These findings suggest that caregiver wellbeing influences desires for future care, potentially resulting in perspectives that are not in‐line with cultural values. As such, culturally tailored interventions are recommended to meet everyday care needs.

